# Supplementary material for: Hierarchical abstraction drives human-like 3-D shape processing in deep learning models
Source: PLoS Comput Biol. 2026 Mar 13;22(3):e1014047. doi: 10.1371/journal.pcbi.1014047 (PMC13052885; doi:10.1371/journal.pcbi.1014047)
Supplement: Attachment — Submitted filename: [PLoS Comp Bio] Response to Reviewers.pdf [file pcbi.1014047.s001.pdf]

PLOS Computational Biology Response to Reviewers

We thank the editors and reviewers for their thoughtful feedback, which substantially improved the manuscript. In the Introduction, we rewrote and streamlined the narrative to better motivate the central question, added missing foundational references, strengthened the scope framing to clarify the paper's biological relevance while avoiding over-general claims, and expanded the neuroscience context on hierarchical processing. In the Methods, we moved the Methods section to follow the Introduction for accessibility, added a new "Training process" subsection reporting full training hyperparameters, data augmentation, and model sizes, clarified the practice-trial design, justified key experimental design choices, and specified how model error bars were computed. In the Results, we added fuller statistical reporting for Experiment 1, extended the confusion-matrix correlation analysis to Experiment 1 to assess human-model alignment beyond accuracy, and clarified the rationale and stimulus-selection procedures in the part-scrambled experiment. We also added new model evidence by evaluating three additional downsampling-based architectures (PointNet++, PointNeXt, and PCT) across all experiments, showing consistently strong human-model correspondence. In the Discussion, we explicitly addressed the training-configuration confound by noting that all models were trained with identical augmentation and that training dropout exceeded the most extreme density-drop condition, refined claims to emphasize that downsampling (rather than attention per se) drives effects within this controlled point-cloud setting, and added discussion of limits and future directions.

Below is the detailed list of modifications we incorporated, based on the comments received.

Reviewer #1

Comment 1

The model performance on the density drop experiment can be closely related to how the model is trained, specifically how does the data augmentation drops the points. In the method section, the authors stated "random point dropout" is used, but details on the dropout ratio are not reported. If the dropout ratio matches the "drop proportion" in the experiment, the model could perform well. Then the question is, if the above is true, such that the performance depends on the training configs, how to interpret the result, such as "Point Transformer is more robust and more similar to human performance". Is it that the model is more robust, or the training config makes it more robust? The authors should discuss the effects of training configs.

The model and training details in the method section are minimal. The authors should add details such as learning rate, epoch, model size, etc.

Response

We thank the reviewer for these valuable comments regarding the training configuration and data augmentation. We have now added a new subsection titled "Training process" under the Modeling methods section, which provides full details on the training setup, including learning rate, number of epochs, model size, optimizer, and data augmentation parameters.

Specifically, all models were trained using the same data augmentation procedures, following the standard implementation of Point Transformer. The random point dropout ratio during training was set to [0, 0.875], meaning that each model was exposed to substantially stronger point dropout than the lowest point-density condition used in our behavioral experiments (with the point dropout ratio 0.8). Therefore, all models were trained under more challenging dropout conditions.

Because both the DGCNN and Point Transformer were trained with identical data augmentation and optimization settings, the differences observed in performance and human-model correspondence reflect architectural differences—particularly the presence of the hierarchical downsampling mechanism—rather than differences in training configuration. We have clarified this point in the revised manuscript. In the discussion, we note that point cloud displays are novel for human participants and differ substantially from the way three-dimensional objects are typically encountered in the natural environment. As a result, data augmentation through variations in point density alone is unlikely to account for human performance. Instead, we suggest that humans may rely on alternative forms of data augmentation, such as active interaction and manipulation of objects, to support the learning of three-dimensional shape representations.

Comment 2

In Experiment 3, are subjects informed that the parts are scrambled? If they are not (though it is close to the DNN setting; DNN doesn't see any instruction), this would be a confusing task. If they are, then it is not a fair comparison to DNNs. The authors should discuss this point

.

Response

No, participants were not informed that the parts were scrambled. Participants were simply asked to identify the object category that best matched each rotating point cloud, as in previous experiments. However, they encountered both scrambled and intact point clouds intermixed with random trial order. This maintains comparability to the DNNs, which also receive no semantic cue or metadata about shape coherence.

Even though the task is more challenging than the previous two experiments, participants still maintained a relatively high performance (over 70% accuracy) on the scrambled point clouds, demonstrating their capabilities in identifying the part-scrambled objects using local shape features, for example, recognizing wing-like shapes to infer the airplane category.

Comment 3

In Fig 8. The examples given in a) and b) are different. Choosing the same instance but scrambling based on DGCNN and Point Transformers can be more informative.

Response

We agree that using the same instance in both (a) and (b) enables a clearer and more direct comparison. We have updated Fig. 8 (now Fig. 9 in the revision) so that (a) and (b) now use the same example instance, with scrambling performed respectively based on DGCNN and Point

Transformer. This change improves the interpretability and fairness of the comparison.

Comment 4

A broader question is whether the labels are still meaningful after scrambling. The authors may get the inspiration from the known texture-shape bias in CNN and human perception. However, I believe part-whole bias is less natural to study compared to other biases. In studies on texture-shape bias (Geirhos, Robert, et al. 2018, should cite in the introduction when the authors talk about CNN uses less global information), the visual concepts they used have a stereotypical texture and shape (e.g. elephant vs cat); from either texture or shape, the subject should be able to identify the visual concept. For the same reason, the design of Baker 2019 et. al. is also valid. However, in the experiment 3 on part-whole, I believe parts only do not contain sufficient information to identify the object, especially given that classes like chair and table are similar. The parts can be just simple simplices.

Response

We cited Geirhos et al (2018) in the intro section. Thanks for pointing out this paper. This is an interesting point about part-whole bias. To ensure that the scrambled stimuli remained interpretable and comparable across humans and models, we specifically selected scrambled point clouds for which the model produced the same category prediction as for the original intact object to measure human judgments. This is the same procedure as used in part scrambling in the paper by Baker et al. 2018.

This selection procedure ensured that, according to the model's own internal representation, the scrambled configuration still contained enough geometric information to support the same categorical decision. Therefore, if humans also achieved high accuracy on these scrambled stimuli, it would indicate that humans and the model rely on largely overlapping features or information sources (likely local shape features maintained in parts) for object recognition. Conversely, the extent of the accuracy drop in human performance reflects how much of the information used by the model is not shared by humans. In the extreme case, if human accuracy remained unchanged after scrambling, it would suggest that humans and the model utilize nearly identical diagnostic features.

In this sense, the reduced meaningfulness of the labels in the scrambled condition is not a flaw but a deliberate methodological probe: it allows us to assess the degree of correspondence in feature use between humans and models that produce the same categorical outputs. Put another way, if humans were not using information that was disrupted by scrambling, their performance would have remained unchanged as was the model performance for these displays.

Finally, despite the difficulty of the part-scrambling experiment, human recognition performance remained reliably above chance (1/6), even for chair and table categories, indicating that local object parts still provide some informative cues to support category identification.

Comment 5

Line 275-279, the authors claim Point Transformer relies more on global structure, because its acc on scrambled inputs is 64% compared to 74% in DGCNN. However, Fig 9. shows a large variability

across categories. It actually looks like Point Transformer is better than DGCNN on 3 over 6 categories. The low relative accuracy is likely due to DGCNN has a surprising perfect accuracy on airplane, which is likely due to the Point Transformer-based scramble of airplane preserving the global shape. This is a huge confounder for making the above conclusion. The experiment design would be cleaner if using the same scrambled inputs on both networks.

Response

We agree with the reviewer that using identical scrambled inputs for both models would constitute a cleaner experimental control. In practice, however, we were unable to identify a single set of scrambled stimuli that simultaneously satisfied this single-set constraint while also yielding 100% accuracy for both models. As a result, we adopted the experimental design reported in the manuscript. Part-scrambling, regardless of whether it is generated from DGCNN or Point Transformer, disrupts the global shape of objects by altering the spatial relations among parts. That said, the reviewer's observation is correct: the observed asymmetry in performance between the two models is largely driven by a single object category, airplane. In this case, wing-like components remain a highly diagnostic local cue for identifying airplanes. DGCNN appears to rely more heavily on this cue, which likely accounts for its unusually high accuracy on the Point Transformer–based scrambled airplane stimuli. While this result is not inconsistent with our broader claims, it does indicate that the average performance difference is disproportionately influenced by one category. In light of this, we have removed the interpretation of the asymmetry result between the two models, and now focus on discussions of human performance for these part-scrambled stimuli.

Comment 6

Line 332-334, the authors claim "Downsampling mechanisms facilitate hierarchical processing within the model, enabling global integration of local shape information and reducing dependence on local features". The evidence from the point density drop experiment does not directly support this claim. The experiment drops points randomly across the whole point cloud. It is unclear whether it disrupts more local features than global shape features. The Experiments 2 and 3 would be more direct support. Similarly, the "Intervention simulation" section is tested on density drop, but should also test other experiments. The authors indeed did those experiments and use the results to compute the correlation between human performance in Fig 12. The results on each task should be reported to show which task differs the most with and without downsampling.

Response

In our point-density drop manipulation, points are removed uniformly at random across the entire object surface. Given that the stimuli in our study are composed of relatively large parts, this procedure produces a distributed thinning of surface sampling rather than removing an entire part or disrupting part configuration. As illustrated in Fig. 3, even at substantial drop rates the object's global structure and the spatial relations among major parts remain largely intact across most conditions, while fine-grained local surface details become less reliably represented.Therefore, we interpret the manipulation as one that disproportionately degrades local geometric cues while preserving the global arrangement.

The intervention results of Experiment 2 have been added to Fig. 11 (now Fig. 12), and the corresponding results and discussion have been incorporated into the "Intervention simulation" section.

Comment 7

Line 363-366, "While it is commonly assumed that the attention mechanism in transformer-based architectures primarily enables better generalization, our results clearly demonstrate that it is actually the Downsampling mechanism driving this effect." This claim should be more specific than general. In general, most of the transformer-based architectures do not have downsampling, but convolution-based architectures have downsampling. And the authors should discuss why this 3d point cloud case is different from other cases on different modalities, such as 2d images, time sequence, etc.

Response

We agree that our original statement was overly broad and could be misinterpreted as a general claim about transformer architectures across modalities. Our findings are specific to the point cloud object recognition setting. In our comparisons, the key contrast was not "transformer vs. convolution," but rather architectures with explicit downsampling versus closely matched variants without downsampling (e.g., Point Transformer vs. Point Transformer noDS; DGCNN+DS vs. DGCNN). Within this controlled setting, the improvement in robustness and human-model alignment consistently tracked the presence of downsampling rather than the use of attention per se. We have narrowed and contextualized the claim in the paper, explicitly stating that it applies to the point cloud recognition task here.

We also agree that transformer-based models in general do not necessarily include downsampling, and many convolutional pipelines do. Regarding modality differences, the point cloud case differs from 2D images or time series in that point clouds are irregular, unordered samples of a surface, and density changes directly alter the availability of local geometric cues (e.g., surface normals, curvature, and neighborhood structure). Without hierarchical sampling and aggregation, models can become more sensitive to local neighborhood statistics and sampling density. In contrast, 2D images and many time-series inputs are already represented on regular grids, where locality and multi-scale structure are naturally supported through fixed receptive fields or pooling operations. Thus, the point cloud setting benefits especially from mechanisms that stabilize representations across variations in sampling density and local neighborhood reliability.

Reviewer #2

Comment 1

Each experiment recruited only a few dozen participants with an average age around 20 years, likely undergraduate students. This narrow demographic range limits generalizability. Including more diverse participants or providing justification for this selection would strengthen the validity of the findings.

Response

We appreciate the reviewer's observation regarding the limited demographic range of our participants. As noted, the participant pool primarily consisted of undergraduate students recruited via the UCLA SONA system, resulting in an average age around 20 years. We acknowledge that this sample may not fully represent the general population. However, shape perception is relatively stable across adulthood and cultures, and the object categories examined in this study are common everyday objects. We therefore expect human performance to generalize to some extent. At the same time, the stimuli and experimental paradigm can be readily extended to other age groups and populations, enabling future work to address important questions about the development and aging-related changes of shape perception. We added this point to the discussion section.

Comment 2

Human participants possess rich visual experience, while the models were trained solely on the ModelNet40 dataset. Future work should test model generalization on more diverse or self-supervised 3D datasets to better approximate human learning conditions.

Response

We appreciate the reviewer's insightful comment regarding the difference in visual experience between human participants and models trained solely on the ModelNet40 dataset. Our goal in this study was to isolate the effects of architectural design on generalization, rather than the influence of training data scale or diversity. Using a controlled and widely adopted benchmark dataset such as ModelNet40 allowed us to systematically compare architectures under identical training conditions, thereby ensuring that observed differences in performance reflect architectural factors rather than dataset variability.

We agree that exposure to more diverse 3D datasets and self-supervised training paradigm could further enhance model generalization and more closely approximate human visual learning. Exploring how such training paradigms interact with architectural design represents an important direction for future research. We added this point to the discussion.

Comment 3

Only two neural architectures were compared. Incorporating additional representative models, such as PointNet++ or recent hybrid graph-transformer architectures, would enhance the robustness and generality of the conclusions.

Response

We further evaluated three downsampling-based point-cloud models (PointNet++, PointNeXt, and PCT) on the same stimuli across all three experiments. Using the same correlation-based analysis, all three models showed strong correspondence with human accuracy patterns. Together with the results from DGCNN+DS and the original Point Transformer, these findings suggest that hierarchical processing enabled by downsampling promotes more human-like 3D shape recognition, and that downsampling-based models align more closely with human performance than non-downsampling variants (DGCNN and Point Transformer noDS).

Comment 4

It's not just the network architecture. I think brain-like neural networks, such as spiking neural networks, are also worth trying, so that we can know whether the results are related to the neuron model or the network structure.

Response

We agree that exploring biologically inspired neuron models, such as spiking neural networks, would be an interesting direction for future work. In the present study, however, our goal was to isolate and examine the architectural mechanisms—specifically, the downsampling process supporting hierarchical abstraction—that give rise to human-like robustness in 3D shape recognition.

While spiking neural networks differ in the neuronal computation model, they would introduce additional dynamics (e.g., temporal coding, spike timing) beyond the architectural features we aim to characterize here. Thus, incorporating such models would require a separate experimental and computational framework, which we consider beyond the current scope. We have added a brief discussion noting that future work could test whether similar hierarchical abstraction mechanisms emerge in spiking or other biologically detailed networks.

Comment 5

The comparisons rely mainly on classification accuracy. It would be valuable to include representational similarity analysis (RSA), confusion matrix correlation, or embedding-space visualization to deepen the understanding of model–human correspondence.

Response

In the original submission, we included a confusion matrix correlation analysis in Experiment 2 (Fig. 8), showing the degree of correspondence between human and model error patterns. Following the reviewer's advice, we have now extended this analysis to Experiments 1 as well. Specifically, we computed Pearson correlations between the off-diagonal elements of the human and model confusion matrices. Consistent with Experiment 2, we found that the Point Transformer exhibited stronger correlations with human error patterns than DGCNN, reinforcing our conclusion that hierarchical abstraction supports human-like representational structure.

Comment 6

While detailed, the manuscript could benefit from greater focus. Streamlining sections with redundant experimental details and emphasizing key theoretical contributions would improve readability and highlight the central message.

Response

We rewrote the introduction section, and removed redundant experimental details, and streamlined the sections.

Comment 7

Although the discussion draws analogies between downsampling and hierarchical visual processing in the human brain, the paper would benefit from citing or connecting more directly to neuroscientific evidence supporting hierarchical abstraction in biological vision.

Response

We included a brief summary from neuroscience studies, and expanded the relevance to neuroscience in the discussion section.

Reviewer #3

Comment 1

Although the work is methodologically thorough and the question is potentially meaningful, the central contribution lies in analyzing model performance differences rather than offering insights into biological mechanisms. The study primarily performs *model–human comparisons* to infer internal properties of deep learning models, but it does not provide new understanding about the neural or computational principles of biological vision. As such, the manuscript reads more as a machine learning study on architectural design than a biological modeling paper, which falls outside the core scope of *PLOS Computational Biology*.

Response

We thank the reviewer for this thoughtful comment. Although a central focus of the paper is the comparison between models and human performance, the work also makes a substantive contribution to the empirical study of the human visual system. To our knowledge, this is the first study to employ point cloud stimuli across a broad range of object categories and a large number of object instances. By systematically manipulating local geometric features and global shape structure, our paradigm provides a well-controlled empirical test that is well suited for examining both the development of shape perception and its impairments. In the revised manuscript, we have clarified and highlighted these contributions to empirical research.

We view this work as a step toward understanding which architectural inductive biases contribute to human-like shape processing, rather than a claim about direct neural correspondence. In this way, the work remains within the scope of PLOS Computational Biology: it connects computational modeling with biological inspiration, providing systematic evidence that architectures motivated by biological vision principles—namely hierarchical, abstraction-based processing—yield models that more faithfully capture human-level robustness.

Comment 2

The title emphasizes “hierarchical abstraction drives…” as if the study provides direct evidence or theoretical grounding for hierarchical abstraction in biological visual systems. For me, it is very hard to link “hierarchical abstraction” and their experiments & results. The title and framing should therefore be reconsidered.

Response

In our manuscript, the phrase “hierarchical abstraction drives human-like 3D shape processing” refers specifically to the idea that computational mechanism adopted in deep learning models support human-like robustness and global shape sensitivity, rather than to biological neural hierarchy per se.

We therefore respectfully prefer to retain the current title, as it accurately reflects our main finding: that hierarchical abstraction mechanisms within deep models are the primary drivers of human-like 3D shape processing performance.

Comment 3

Introduction flow - The Introduction reads as a sequence of descriptive paragraphs rather than a clear argument that motivates the key scientific question.

Response

We have now rewritten the introduction section to better motivate the work**.**

Comment 4

The authors missed a lot of method details. e.g., It is unclear whether the stimuli in the practice trials differed in design or difficulty from those in the main task. The rationale for this distinction should be clarified.

Response

We thank the reviewer for this helpful comment. In the original submission, the full Methods section was placed after the Discussion, following the PLOS Computational Biology format. We realize this placement may have made it difficult to locate specific methodological details. In the revised version, we have moved the Methods section to immediately follow the Introduction for greater clarity and accessibility.

Regarding the reviewer's specific question, the practice trial used the same type of rotating point-cloud stimulus as in the main experiment but featured a single object (a plant) that did not appear in any subsequent trials. The purpose of the practice trial was only to familiarize participants with the task format and ensure understanding of the response procedure; its design and difficulty were not otherwise manipulated. We have clarified this information in the General procedure section.

Comment 5

We need to see more statistical reports. e.g., Figure 4 and related results lack statistical details. Given the multiple conditions (point density, orientation), the authors should report relevant statistics (e.g., ANOVA main effects and interactions) and show *p* and *t* values directly on the figure. For the upright vs. inverted comparison, a proper test of orientation effects is warranted.

Response

We appreciate the reviewer’s suggestion to include more detailed statistical analyses. To improve the completeness of the report, we have now included a paragraph in the Results section of Experiment 1 providing the corresponding statistical analyses.

We have chosen not to include p and t values directly on Figure 4, as this figure is intended to illustrate the overall trends in human and model performance rather than to highlight inferential statistics. Instead, all statistical results are now explicitly reported in the main text of the Results section.

Comment 6

The manuscript does not explain how model error bars were derived (across random seeds? test batches?). This is essential for interpreting Figures 4–9 and assessing model variability.

Response

Thank you for pointing this out. We have now clarified the derivation of the model error bars in the figure captions. Specifically, model error bars represent 95% confidence intervals computed across stimuli within each experimental condition, whereas human error bars are computed across participants.

Comment 7

In Fig 9, the DGCNN model shows higher accuracy for “scrambled” than for intact point clouds, which is counterintuitive and undermines the claim of human-like correspondence. The authors should address why this occurred and what it implies for their interpretation.

Response

We intentionally selected scrambled stimuli for which DGCNN correctly identified the target category. No such selection was applied to the intact stimuli. We have clarified this distinction in the revised Methods section for Experiment 3.

Comment 8

The authors cite their own prior work on texture bias but omit the foundational *Geirhos et al., 2019 ICLR* paper, which is essential to contextualize this topic. Key references must be included.

Response

Thank you. We included this paper.

Comment 9

The authors should consider discussing recent findings (e.g., O'Connell et al., 2025, *Open Mind*) showing that multi-view representations—not simply hierarchical abstraction—may explain improved model–human alignment in 3D perception.

Response

We appreciate the suggestion to consider O'Connell et al. (2025, Open Mind). That paper shows that improved model-human alignment in certain 3D perception tasks can be explained by multi-view representations induced by training objectives that explicitly encourage viewpoint-consistent embeddings. However, our study is aimed at a different explanatory level. We hold training broadly constant and instead use behavioral experiments plus targeted ablations to ask which architectural components in point cloud models account for human-like robustness patterns across distinct manipulations. Our main goal is not to explain alignment via multi-view objectives, but to identify the computational mechanisms that distinguish model families under matched conditions.

We therefore view O'Connell et al.'s results as complementary rather than directly bearing on the central claims of the present paper: they emphasize how multi-view learning objectives can drive alignment, whereas we emphasize how architectural operations shape the representations that support human-like behavior in point-cloud recognition.

Comment 10

Why is *point density* treated as a within-subject factor while *orientation* is across-subject? This design choice should be justified.

Response

We thank the reviewer for this insightful question. The decision to treat point density as a within-subject factor and orientation as a between-subject factor was based on both methodological and practical considerations.

Manipulating orientation within subjects would have required presenting the same objects in both upright and inverted positions, which could produce memory and recognition biases. Participants might recognize the inverted object by recalling its upright counterpart rather than relying on perceptual processing.

We recognize that an alternative design—assigning distinct sets of objects to upright and inverted conditions—could also control for cross-condition familiarity. However, we prioritized a between-subject design for orientation to ensure clear interpretability of orientation effects and to maintain a manageable experiment length. We have added this rationale to the revised Methods section for clarity.

Comment 11

The statement “The stimuli were selected from the test set… to ensure a fair comparison” is unclear—why does choosing from the test set guarantee fairness between humans and models?

Response

We thank the reviewer for pointing out this ambiguity. In the original submission, our intention in selecting stimuli from the test set of the ModelNet40 dataset was to avoid any overlap with the data used for model training, thereby ensuring that both humans and models were evaluated on identical, unseen stimuli. This procedure guarantees that the model–human comparison is fair: humans and models were both tested on the same set of previously unseen objects, and model performance was not influenced by prior exposure to the training examples.

Comment 12

It would be informative to report the parameter counts for DGCNN and Point Transformer to clarify whether performance differences might stem from model capacity rather than architecture alone.

Response

We thank the reviewer for this helpful suggestion. We have now added a row reporting the parameter counts of each model in Table 1 of the revised manuscript. Specifically, DGCNN contains 1.81 million parameters, while the Point Transformer has 9.58 million parameters.

To address the reviewer’s concern about whether the observed performance differences might arise from model capacity rather than architecture, we note that in our ablation study, we replaced the Transition Down layers in the Point Transformer with MLP layers so that the resulting no-downsampling variant had the same total number of parameters as the original Point Transformer. Despite identical parameter counts, this variant exhibited reduced robustness and weaker global shape sensitivity, indicating that the key difference arises from the architectural mechanism of hierarchical downsampling, not from model size.

Conversely, when we added downsampling layers to the DGCNN architecture, the modified model displayed enhanced robustness and a clearer global shape bias, consistent with our conclusion that hierarchical abstraction, rather than parameter count, drives human-like performance.
